# Transmembrane TNF-α promotes activation-induced cell death by forward and reverse signaling

**DOI:** 10.18632/oncotarget.19124

**Published:** 2017-07-10

**Authors:** Meng Zhang, Jing Wang, Lingwei Jia, Jin Huang, Cheng He, Fuqing Hu, Lifei Yuan, Guihua Wang, Mingxia Yu, Zhuoya Li

**Affiliations:** ^1^ Department of Immunology, Basic Medical College, Tongji Medical College, Huazhong University of Science and Technology, Wuhan 430030, P.R. China; ^2^ Molecular Medical Center, Tongji Hospital, Huazhong University of Science and Technology, Wuhan 430030, P.R. China

**Keywords:** activation-induced cell death, transmembrane TNF-α, secretory TNF-α, reverse signaling, apoptosis

## Abstract

Secretory tumor necrosis factor-alpha (sTNF-α) is known to mediate activation- induced cell death (AICD). However, the role of tmTNF-α in AICD is still obscure. Here, we demonstrated that tmTNF-α expression significantly increased accompanied with enhanced apoptosis during AICD in Jurkat and primary human T cells. Knockdown or enhancement of tmTNF-α expression in activated T cells suppressed or promoted AICD, respectively. Treatment of activated T cells with exogenous tmTNF-α significantly augmented AICD, indicating that tmTNF-α as an effector molecule mediates AICD. As tmTNF-α can function as a receptor, an anti-TNF-α polyclonal antibody was used to trigger reverse signaling of tmTNF-α. This antibody treatment upregulated the expression of Fas ligand, TNF-related apoptosis-inducing ligand and tmTNF-α to amplify AICD, and promoted activated T cells expressing death receptor 4, TNF receptor (TNFR) 1 and TNFR2 to enhance their sensitivity to AICD. Knockdown of TNFR1 or TNFR2 expression totally blocked tmTNF-α reverse signaling increased sensitivity to sTNF-α- or tmTNF-α-mediated AICD, respectively. Our results indicate that tmTNF-α functions as a death ligand in mediation of AICD and as a receptor in sensitization of activated T cells to AICD. Targeting tmTNF-α in activated T cells may be helpful in facilitating AICD for treatment of autoimmune diseases.

## INTRODUCTION

Activation-induced cell death (AICD) refers to activated T cells expressing death receptors and ligands that triggers caspase cascade, inducing suicide and fratricide by apoptosis upon persistent or repeated stimulation of their T cell receptor (TCR). AICD plays a critical role in maintenance of peripheral immune tolerance and protection against autoimmune diseases through deletion of overactivated or autoreactive T cells in the periphery [1]. AICD is induced in activated T lymphocytes via the interaction of death receptors and their cognate ligands, including Fas (CD95)/FasL, tumor necrosis factor-alpha (TNF-α)/TNF Receptor 1 (TNFR1), and TNF-related apoptosis-inducing ligand (TRAIL)/Death receptor 4 or 5 (DR4/DR5). Activation of these death receptors results in recruitment of TRADD, or/and FADD, and caspase 8, forming death-inducing signaling complexes and leading to apoptosis of activated T lymphocytes [2–5].

TNF-α exerts biological functions in two forms: 26kD transmembrane TNF-α (tmTNF-α) and 17kD secretory TNF-α (sTNF-α). tmTNF-α can be hydrolyzed by TNF alpha converting enzyme (TACE) to yield sTNF-α [6, 7]. These two forms of TNF-α display their activities through TNFR1 and TNFR2. Although sTNF-α binds two types of TNFR, it dissociates with TNFR2 rapidly. Therefore, the most functions of sTNF-α is through TNFR1. In contrast, tmTNF-α binds the both types of TNFR stably, thus it binds TNFR2 with higher affinity than sTNF-α and is a primary ligand for TNFR2 [8].

Although Fas/FasL-mediated apoptosis is a major contributor to ACID, TNF-α is also one of effector molecules in AICD. It has been reported that sTNF-α can mediate mature T-cell receptor-induced apoptosis, inducing the death of most CD8^+^ T cells, whereas FasL mediates the death of most CD4^+^ T cells [4]. In addition, sTNF-α has also been shown to participate in apoptosis of CD4^+^ T cell [9].

tmTNF-α acts not only as a ligand, transmitting ‘forward signaling’ to target cell via TNFR, but also as a receptor, delivering ‘reverse signaling’ to tmTNF-α expressing cell via itself [10–12]. It has been reported that infliximab, an monoclonal antibody binding to both forms of TNF-α, induced lamina propia and peripheral lymphocytes apoptosis [13–15], suggesting that tmTNF-α may be involved in AICD via reverse signaling. However, etanercept, a soluble TNFR2-Fc fusion protein, failed to induce apoptosis of activated lymphocytes [14]. The function of tmTNF-α in AICD still remains to be elucidated.

Our previous study demonstrated that tmTNF-α increases NK-mediated cytotoxicity through upregulation of multiple cytotoxic effector molecules including perforin, granzyme B, FasL and both forms of TNF-α via reverse signaling [11]. We assumed that tmTNF-α may also play a role in AICD through its forward and reverse signaling. To test this hypothesis, we investigated the changes of endogenous tmTNF-α in AICD, and the impact of down- or up-regulation of tmTNF-α expression on AICD. We used a polyclonal antibody to trigger reverse signaling of tmTNF-α in T cells and observed its effect on AICD. We found that tmTNF-α mediated AICD via forward signaling and sensitized activated T cells to apoptosis via reverse signaling.

## RESULTS

### Elevation of tmTNF-α expression in activated T cells in AICD

**Figure 1:**
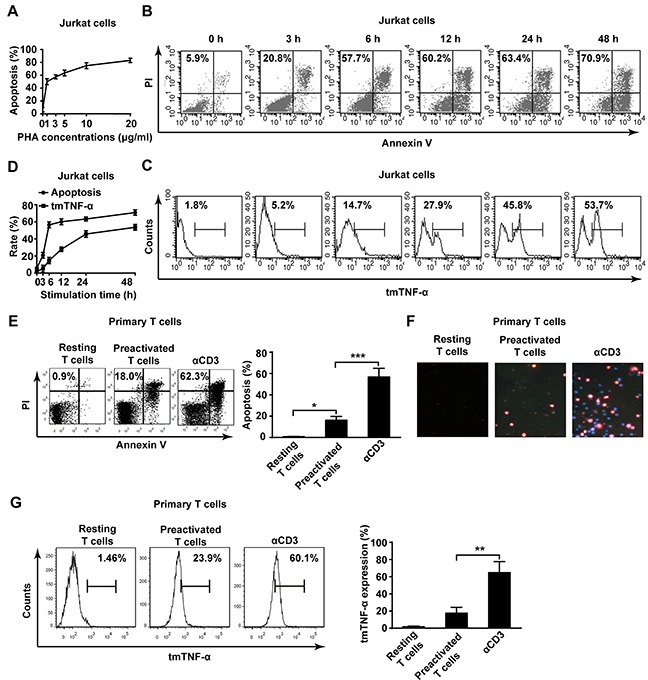
Elevation of tmTNF-α expression in AICD **(A)** Jurkat cells were activated with PHA-P for 24 h in indicated doses and the apoptosis was evaluated by Annexin V/PI. **(B, C, D)** Jurkat cells were stimulated with 5 μg/ml PHA for indicated time points. The apoptosis (B) and tmTNF-α expression (C) were analyzed by flow cytometry. The kinetic curves of apoptosis rate and tmTNF-α expression were shown in the panel D. **(E, F, G)** Primary human T cells were preactivated by PHA (5 μg/ml) for 3 days and cultured in the presence of IL-2 (50 U/ml) for 7 days. The preactivated T cells were restimulated for 24 h with plate-coated αCD3 (OKT3, 10 μg/ml). The apoptosis was measured by Annexin V/PI (E, left) and its quantitative analysis was shown in the histogram (E, right). Hochest/PI double staining in situ for apoptosis (F). The tmTNF-α expression was determined by flow cytometry (G, left) and its quantitative analysis was shown in the histogram (G, right). All the quantitative data represent means ± S.D. of at least three independent experiments. *p<0.05, ** *p*<0.01, ***p<0.001.

To test whether there is an association of tmTNF-α with AICD, a human leukemia T cell line, Jurkat cell, was stimulated with PHA to induce AICD [16–18]. We found that PHA stimulation induced apoptosis of Jurkat cells in a dose-dependent manner. The apoptosis rate of Jurkat cells was elevated gradually with increasing doses of PHA, and reached 63% and 83% in a dose of 5 μg/ml or 20 μg/ml, respectively (Figure 1A). We chose 5 μg/ml of PHA to observe the kinetics of tmTNF-α expression and AICD. As shown in Figure 1B-1D, apoptosis rate increased gradually with the time after PHA stimulation, which was accompanied with raised tmTNF-α expression, indicating an association of tmTNF-α with AICD. As tmTNF-α expression and apoptosis rate reached nearly a plateau 24 h after stimulation with PHA, we chose 5 μg/ml of PHA to activate Jurkat cells for 24 h to induce AICD in the following experiments.

In addition, the same phenomenon was observed in another AICD model in that primary human T cells isolated from PBMC were preactivated with PHA (5 μg/ml), then re-stimulated with αCD3 mAb to induce AICD [19, 20]. The apoptosis of T cells was significantly increased (over 60%) in AICD upon αCD3 restimulation detected by both Annexin V/PI (Figure 1E) and Hochest/PI double staining (Figure 1F). tmTNF-α expression was also markedly enhanced in primary T cells after restimulation (Figure 1G). These results strongly suggest that tmTNF-α expressed in activated T cells may be involved in AICD.

### tmTNF-α is involved in AICD

**Figure 2:**
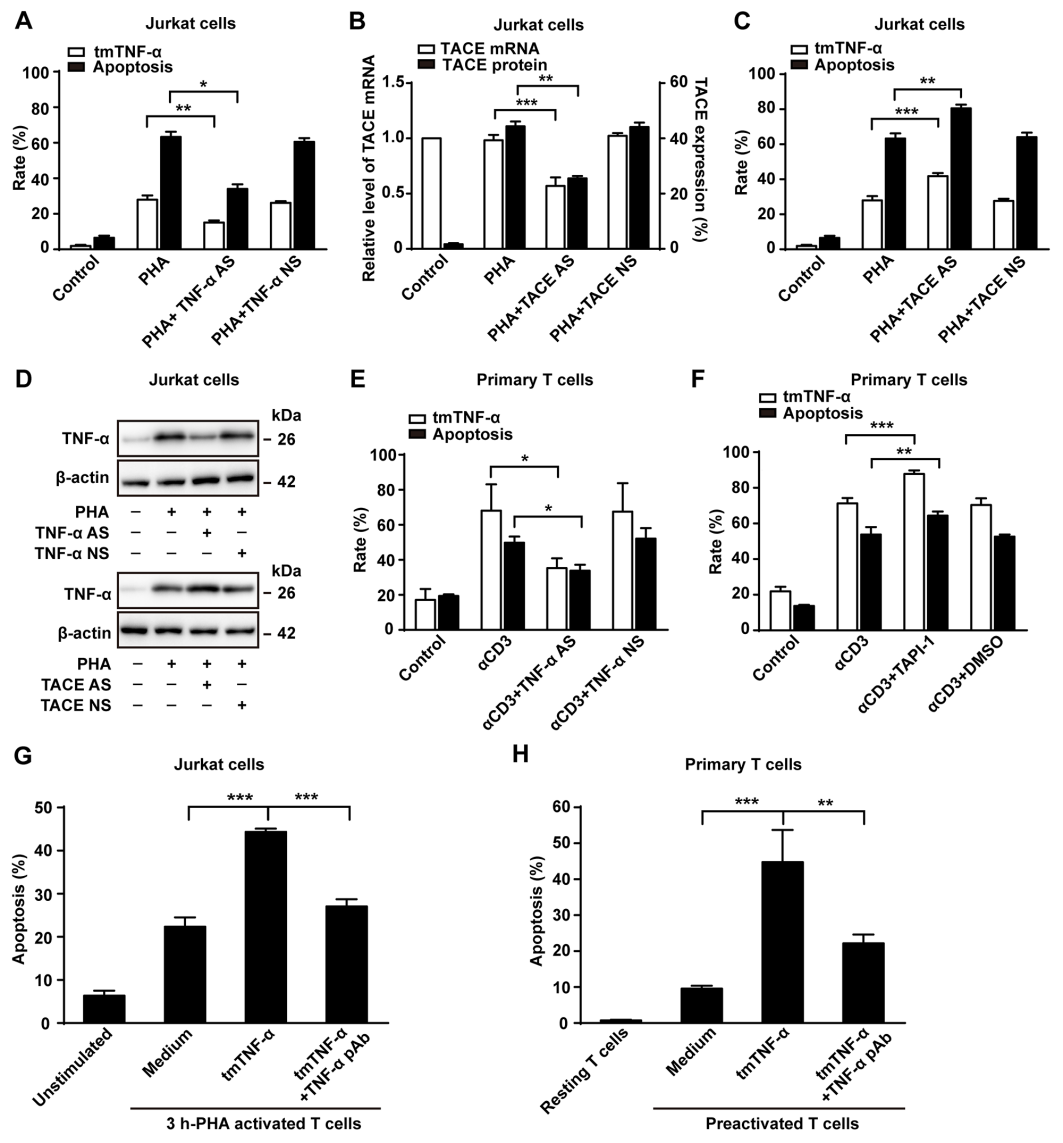
tmTNF-α is involved in AICD **(A, B, C, D)** Jurkat cells transfected with 10 μM of TNF-α AS, TACE AS or NS for 48 h were treated with PHA-P (5 μg/ml) for 24 h. Apoptosis rate (A,C) was evaluated by Annexin V/PI and expression of tmTNF-α (A,C) and TACE (B) on the cell surface was analyzed by flow cytometry. Relative levels of TACE mRNA was detected by Real time qPCR (B). Western blot analysis of tmTNF-α expression (D). **(E, F)** PHA-preactivated primary T cells were transfected with 10 μM of TNF-α AS or NS for 48 h, and then treated with αCD3 (10 μg/ml) for 24 h. A TACE inhibitor TAPI-1 (10 μM) was added simultaneously with αCD3 treatment, and vehicle DMSO served as a control. Apoptosis was evaluated by Annexin V/PI and tmTNF-α expression was detected by flow cytometry. **(G, H)** tmTNF-α overexpressing 24 h-PHA activated and fixed Jurkat cells were cocultured with 3 h-PHA activated Jurkat cells at an effector/target ratio of 10:1 for 48 h (G). For primary T cells, tmTNF-α overexpressing, αCD3-restimulated and fixed T cells were co-cultured with PHA-preactivated T cells (preactivated T) at an effector/target ratio of 10:1 for 48h (H). For neutralization of tmTNF-α, effector cells were treated with TNF-α pAb for 30 min and then washed prior to the addition to the target cells. Apoptosis was determined by the Annexin V/PI. All the quantitative data represent means ± S.D. of at least three independent experiments. *p<0.05, **p<0.01, ***p<0.001.

In order to verify the involvement of tmTNF-α in AICD, we used two methods: knockdown of TNF-α expression by TNF-α AS or increase of tmTNF-α expression by silence of TACE expression to suppress the cleavage of tmTNF-α into sTNF-α. As expected, transfection of TNF-α AS inhibited tmTNF-α expression in activated Jurkat cells (Figure 2A and 2D), and the apoptosis rate was notably decreased consequently (Figure 2A). In contrast, transfection of TACE AS, which suppressed TACE mRNA transcription and protein expression (Figure 2B), markedly increased tmTNF-α expression (Figure 2C and 2D). Consequently, the apoptosis rate was significantly enhanced (*p*<0.01), compared with PHA alone treatment (Figure 2C). The similar results were observed in primary T cells treated with either TNF AS or a TACE inhibitor TAPI-1 (Figure 2E and 2F). Of note, PHA alone did not affect TACE mRNA transcription, but significantly increased TACE expression on the cell surface (Figure 2B). These data suggested that tmTNF-α expressed in activated T cells may be an effector molecule to induce AICD.

To test the possibility of tmTNF-α-mediated AICD, we observed whether exogenous tmTNF-α could induce apoptosis of activated T cells. tmTNF-α highly expressing (45.8%) Jurkat cells activated by PHA for 24 h were fixed with 1% paraformaldehyde and used as tmTNF-α-bearing effector cells. To exclude the effect of endogenous tmTNF-α in activated T cells, we used Jurkat cells activated by PHA for 3 h as target cells, because this time point was early stage of AICD at which the apoptosis rate was lower (20.8%) and the activated T cells expressed very low level (5.21%) of tmTNF-α. We co-cultured effector cells and target cells at a ratio of 10:1 for 48 h followed by the Annexin V/PI detection and found that tmTNF-α overexpressing Jurkat cells induced significant apoptosis of 3 h-PHA activated T cells (*p*<0.001). Neutralizing tmTNF-α in fixed effector cells by a specific antibody evidently blocked tmTNF-α-mediated apoptosis in 3 h-PHA activated T cells (Figure 2G). For primary T cells, tmTNF-α overexpressing T cells restimulated with αCD3 for 24 h were fixed with 1% paraformaldehyde and cocultured for 48 h with PHA-preactivated T cells. In line with the results in Jurkat cells, tmTNF-α did induce apoptosis in preactivated T cells (Figure 2H). Our results indicate that tmTNF-α-mediated apoptosis contributes to AICD via forward signaling, as tmTNF-α functioned as a ligand in this case.

### sTNF-α participates in AICD

**Figure 3:**
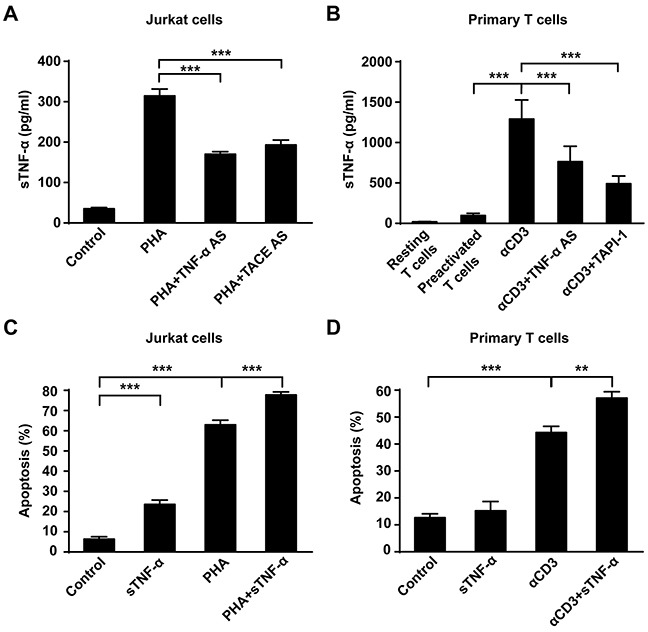
sTNF-α participates in AICD **(A, B)** Jurkat cells transfected with 10 μM of TNF-α AS or TACE AS for 48 h were stimulated with PHA-P (5 μg/ml) for 24 h. For primary T cells, PHA-preactivated T cells transfected with TNF-α AS for 48 h were restimulated with αCD3 (10 μg/ml) for 24 h; or PHA-preactivated T cells simultaneously treated with TAPI-1 (10 μM) and αCD3 (10 μg/ml) for 24 h. Concentration of sTNF-α in supernatants was detected by ELISA. **(C, D)** Recombinant human sTNF-α (50U/ml) was incubated with PHA-P activated Jurkat cells or αCD3-restimulated primary T cells for 24 h and the apoptosis was detected by Annexin V/PI. Non-stimulated Jurkat T cells or preactivated primary T cells served as a control. All the quantitative data represent means ± S.D. of at least three independent experiments. ** *p*<0.01, *** *p*<0.001.

As mentioned above, downregulation of tmTNF-α expression decreased apoptosis rate, while upregulation of tmTNF-α expression increased apoptosis rate. However, in these two cases, sTNF-α in the culture supernatant declined significantly, compared with PHA alone treatment either in Jurkat cells or in primary T cells (Figure 3A and 3B). To investigate whether sTNF-α participate in AICD, commercial sTNF-α (50U/ml) was added and cultured with Jurkat cells for 24 h. As shown in Figure 3C, sTNF-α exerted cytotoxicity not only to unstimulated Jurkat cells but also to PHA-activated T cells, compared with control or PHA alone treatment (*p*<0.001). However, sTNF-α treatment could only boost AICD in primary T cells reactivated with αCD3 but not in PHA-preactivated T cells (Figure 3D), suggesting that sTNF-α participates in AICD.

### tmTNF-α-mediated reverse signaling enhances AICD

**Figure 4:**
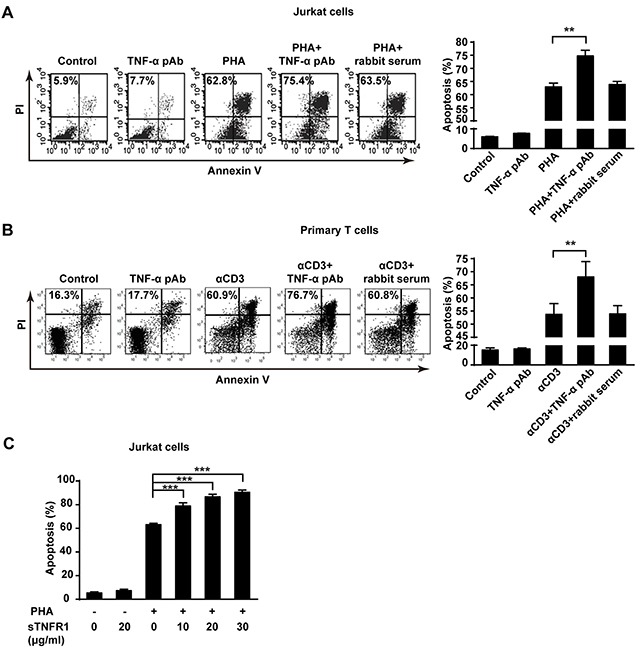
The reverse signaling of tmTNF-α enhances AICD **(A, C)** Jurkat cells were incubated with PHA-P (5 μg/ml), TNF-α pAb (1:100), sTNFR1 or with both PHA-P and TNF-α pAb, or PHA-P and sTNFR1 in indicated concentrations for 24 h. Apoptosis was detected by Annexin V/PI. **(B)** PHA preactivated primary T cells were stimulated with αCD3 (10 μg/ml), TNF-α pAb, or both for 24 h. Apoptosis was detected by Annexin V/PI. Rabbit serum served as a control. All the quantitative data represent means ± S.D. of at least three independent experiments. **p<0.01, ***p<0.001.

In addition to mediation of apoptosis as a ligand in AICD, tmTNF-α may also function as a receptor in promotion of AICD. To test this hypothesis, anti-TNF-α polyclonal antibody (TNF-α pAb) and soluble TNFR1 (sTNFR1) were used to trigger reverse signaling of tmTNF-α, respectively. Indeed, TNF-α pAb augmented apoptosis in PHA activated Jurkat cells (*p*<0.01), compared with PHA alone treatment (Figure 4A). Neither TNF-α pAb alone could induce apoptosis in Jurkat cells, nor normal rabbit serum had influence on AICD, indicating that TNF-α pAb could activate the reverse signaling of tmTNF-α to amplify AICD, rather than directly induce apoptosis. This enhancement effect was further confirmed in primary T cell AICD model, showing an increased apoptosis in αCD3 re-stimulated T cells induced by TNF-α pAb (Figure 4B).

To confirm that tmTNF-α enhances AICD via reverse signaling, we used another activator sTNFR1 to initiate the reverse signaling of tmTNF-α in different concentrations. Similarly, treatment with sTNFR1 led to an obvious increase in apoptosis of PHA activated Jurkat cells in a dose-dependent manner (*p*<0.001) (Figure 4C). These data suggest that tmTNF-α-mediated reverse signaling may increase sensitivity of activated T cells to AICD.

### tmTNF-α-mediated reverse signaling promotes AICD through upregulation of FasL/Fas and TRAIL/DR4

**Figure 5:**
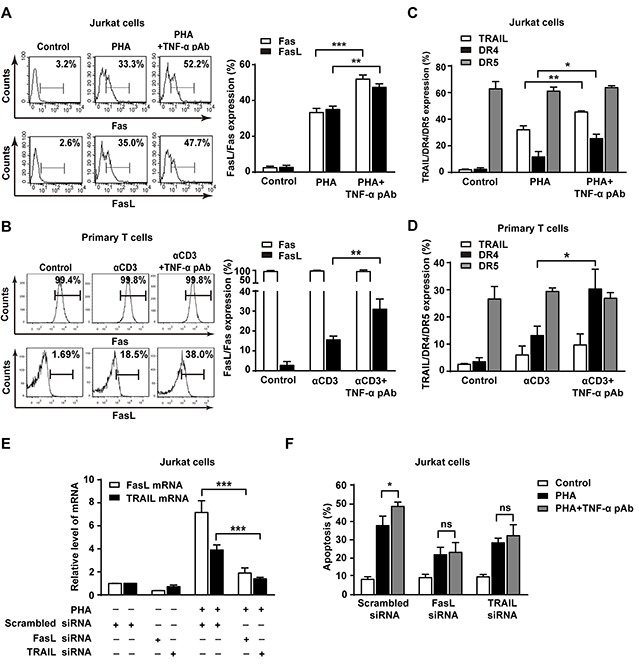
The reverse signaling of tmTNF-α promotes AICD through upregulating FasL/Fas and TRAIL/DR4 **(A, B, C, D)** Jurkat cells were simultaneously stimulated with PHA-P (5 μg/ml) and TNF-α pAb (1:100) for 24 h. PHA-preactivated primary T cells were restimulated with αCD3 (10 μg/ml) and TNF-α pAb for 24 h. Expression of FasL/Fas (A, B) and TRAIL/DR4/DR5 (C, D) was detected by flow cytometry. **(E, F)** Jurkat cells transfected with 100 nM siRNA for FasL or TRAIL for 48 h were activated with PHA-P (5 μg/ml) in the absence or presence of TNF-α pAb for 24 h. The mRNA levels of FasL and TRAIL were determined by Real-time qPCR (E) and the apoptosis was detected by Annexin V/PI (F). All the quantitative data represent means ± S.D. of at least three independent experiments. *p<0.05, **p<0.01, ***p<0.001. ns: no significance.

Death receptors and its cognate ligands such as FasL/Fas and TRAIL/DR4/5 are involved in AICD [21]. We hypothesized that the reverse signaling of tmTNF-α may enable to upregulate the expression of FasL/Fas, TRAIL/DR4/5 to promote AICD. To test this idea, the expression of FasL/Fas and TRAIL/DR4/5 was detected in both AICD models by flow cytometry. As expected, treatment with TNF-α pAb to trigger tmTNF-α-mediated reverse signaling significantly upregulated expression of FasL and Fas in PHA activated Jurkat cells (Figure 5A). But in primary T cell AICD model, FasL was evidently increased by TNF-α pAb treatment and Fas expression remained unchanged at very high levels, similar to that in PHA-preactivated T cells and αCD3 retactivated T cells (Figure 5B). These results indicate that the reverse signaling of tmTNF-α promotes the expression of FasL and/or Fas that are a pair of major contributors to AICD. In addition, TNF-α pAb could greatly enhance expression of TRAIL and DR4, but not DR5, in PHA activated Jurkat cells (Figure 5C). However, in primary T cell AICD model, the expression of DR4, but not TRAIL and DR5, was markedly upregulated by TNF-α pAb treatment, although TRAIL was slightly increased without statistical significance (Figure 5D).

To further explore whether tmTNF-α-mediated reverse signaling promotes AICD via upregulation of these death ligands, we used siRNA to silence of FasL and TRAIL expression, respectively. As expected, knockdown of FasL and TRAIL expression (Figure 5E) not only inhibited PHA-induced AICD, but also totally abolished the enhanced effect of TNF-α pAb on the apoptosis (Figure 5F). The data suggest that reverse signaling of tmTNF-α increases the sensitivity of activated T cells to AICD through upregulating the expression of FasL/Fas and TRAIL/DR4.

### tmTNF-α-mediated reverse signaling enhances the sensitivity of activated T cells to TNF-α-induced AICD by upregulating TNFR1 and TNFR2

**Figure 6:**
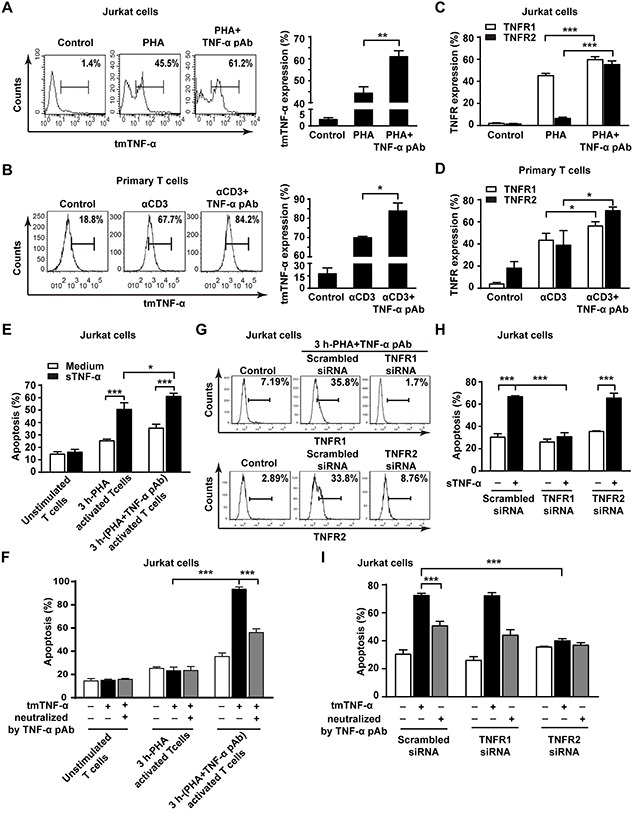
The reverse signaling of tmTNF-α enhances the sensitivity of T cells to TNF-α-induced AICD through upregulating TNFR **(A, B, C, D)** Jurkat cells were stimulated with PHA-P (5 μg/ml) in the absence or presence of TNF-α pAb (1:100) for 24 h. PHA preactivated T cells were reactivated by αCD3 (10 μg/ml) with or without TNF-α pAb (1:100) for 24 h. The expression of tmTNF-α (A, B) and two types of TNFR (C, D) was detected by flow cytometry. **(E, F)** Jurkat cells activated for 3 h by PHA-P (5 μg/ml) with or without TNF-α pAb were incubated for 4 h with sTNF-α (50 U/ml) or tmTNF-α stably transfected and fixed NIH3T3 cells at an effector/target ratio of 10:1. The apoptosis was detected by Annexin V/PI. **(G, H, I)** Jurkat cells transfected with 100 nM siRNA for TNFR1 or TNFR2 for 48 h were activated by PHA-P (5 μg/ml) with TNF-α pAb for 3 h, followed by incubation for 4 h with sTNF-α (50 U/ml) or tmTNF-α stably transfected and fixed NIH3T3 cells at an effector/target ratio of 10:1. The expression of TNFR1 and TNFR2 was analyzed by flow cytometry (G) and the apoptosis was detected by Annexin V/PI (H, I). For neutralization of tmTNF-α, the effector cells were treated with TNF-α pAb for 30 min prior to the addition to the target cells. All the quantitative data represent means ± S.D. of at least three independent experiments. *p<0.05, ** *p*<0.01, *** *p*<0.001.

AICD can be mediated by TNFR1 and TNFR2 [4, 22]. It is possible that the reverse signaling of tmTNF-α may increase tmTNF-α expression by a positive feedback to amplify AICD and upregulate the expression of TNFR1 and TNFR2 to enhance the sensitivity of activated T cells to TNF-α-mediated AICD. To test this hypothesis, we determined the expression of tmTNF-α and two types of TNFR in both AICD models after treatment with TNF-α pAb. As expected, tmTNF-α expression was dramatically increased in PHA-activated Jurkat cells and in αCD3-reactivated primary T cells by treatment with TNF-α pAb (Figure 6A and 6B). Interestingly, the expression of both TNFR1 and TNFR2 increased obviously by TNF-α pAb treatment in both AICD models (Figure 6C and 6D).

To observe whether upregulation of TNFR expression by tmTNF-α-mediated reverse signaling could increase the sensitivity of activated T cells to both forms of TNF-α-induced apoptosis, we compared TNF-α-mediated cytotoxicity to 3 h-PHA-activated Jurkat cells in the absence and presence of TNF-α pAb. The fixed TNF-α stably transfected NIH3T3 cells in that FasL is not expressed [23]were used as tmTNF-α-bearing effector cells. Indeed, either sTNF-α- or tmTNF-α-mediated apoptosis was significantly enhanced even in 4 hours to 3 h-PHA-activated Jurkat cells with activation of reverse signaling of tmTNF-α, in which two type of TNFR were upregulated (Figure 6G), compared with the cytotoxicity of both forms of TNF-α to those T cells without activation of tmTNF-α reverse signaling (Figure 6E and 6F). In contrast, knockdown of TNFR1 expression (Figure 6G) totally blocked the increased sensitivity induced by tmTNF-α reverse signaling to sTNF-α-, but not to tmTNF-α-mediated apoptosis (Figure 6H), while knockdown of TNFR2 expression (Figure 6G) entirely abolished the increased sensitivity to tmTNF-α-, but not to sTNF-α-induced apoptosis (Figure 6I). The data strongly indicate that tmTNF-α-mediated reverse signaling increases the sensitivity of activated T cells to sTNF-α-induced apoptosis via upregulation of TNFR1 expression and to tmTNF-α- induced apoptosis via upregulation of TNFR2 expression.

## DISCUSSION

**Figure 7:**
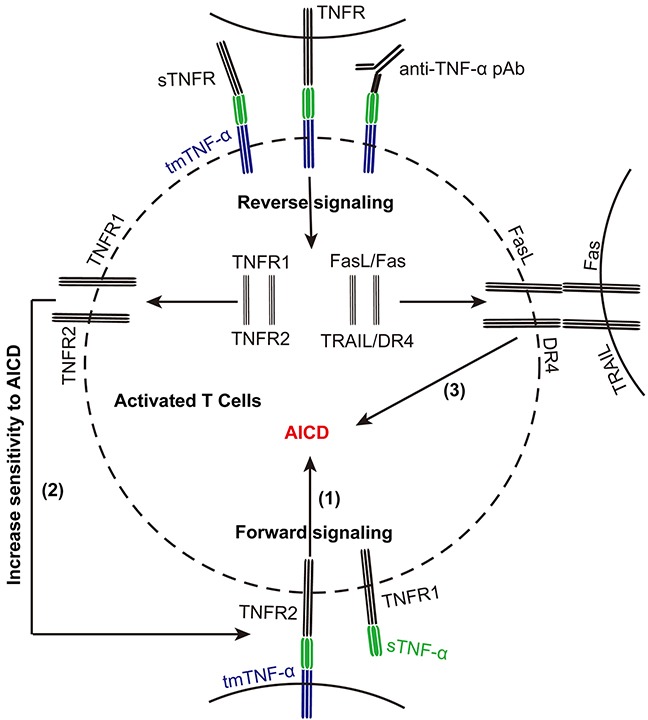
tmTNF-α promotes AICD via forward and reverse signaling tmTNF-α expression is upregulated in activated T cells during AICD. tmTNF-α as a death ligand mediates apoptosis of activated T cells (1). Meanwhile, tmTNF-α as a receptor increases the sensitivity of activated T cells to sTNF-α- or tmTNF-α-induced apoptosis through upregulating TNFR1 or TNFR2, respectively (2). It can also augment the expression of FasL/Fas and TRAIL/DR4 to increase the sensitivity of activated T cells to AICD via reverse signaling (3).

In the present study, we found that tmTNF-α expression in activated T cells was upregulated, accompanied with enhanced apoptosis. This intramembrane molecule functioned not only as a ligand in mediation of AICD in T cells, similar to its soluble form, but also as a receptor on activated T cells in promoting them to express FasL/Fas, TRAIL/DR4 and tmTNF-α/TNFR to increase their sensitivity to AICD (Figure 7).

We found originally that tmTNF-α expression is significantly upregulated in activated T cells, which is associated with increased apoptosis either in PHA-induced AICD in Jurkat cells or in αCD3 reactivation-induced AICD in primary human T cells. Although TNF-α and TNFR deficient mice show normal AICD [24], FasL/Fas and other effector molecules may compensate in this case. In addition, lpr/lpr or gld/gld mice with spontaneous mutations in the fas gene or the fas ligand gene cannot abolish AICD [4, 25, 26], suggesting that other death-inducing molecules including TNF-α are involved in AICD. Our results showed that changing expression levels of tmTNF-α in activated T cells affected AICD consequently, as increasing tmTNF-α expression by TACE AS to suppress the cleavage of this transmembrane molecule led to an enhancement of AICD. On the contrary, inhibiting tmTNF-α expression by TNF-α AS resulted in a decrease of AICD. This phenomenon suggests that tmTNF-α may function as a death ligand in AICD. This is supported by our evidence that tmTNF-α overexpressing, fixed activated T cells could obviously increase apoptosis in both 3 h-PHA activated Jurkat T cells and PHA-preactivated primary T cells and this tmTNF-α-mediated AICD could be blocked by neutralization of tmTNF-α on fixed activated T cells with a specific antibody. As tmTNF-α-mediated apoptosis in activated T cells was prohibited by siTNFR2, but not by siTNFR1, it is likely that tmTNF-α overexpressed on activated T cells, as a death ligand, mediates AICD via TNFR2.

Of note, TACE is a key enzyme in regulation of tmTNF-α processing. We found that PHA increased TACE expression on the cell surface of activated T cells, but did not affect TACE transcription. It has been reported that PHA stimulation leads to the phosphorylation of ERK [27] that mediates phosphorylation of Thr735 in TACE, inducing translocation of TACE to the cell surface [28, 29]. Therefore, PHA-induced increase of TACE expression is due to promotion of translocation of TACE to the cell surface rather than TACE production.

As activated T cells not only express tmTNF-α, but also express TNFR1 and TNFR2 whose expression is strictly regulated, it is possible that tmTNF-α binds TNFR transmitting dual signaling to TNFR-carrying neighboring activated T cells and to tmTNF-α-bearing activated T cells simultaneously. Our results demonstrated that activation of reverse signaling of tmTNF-α by TNF-α pAb or sTNFR1 significantly increased AICD in both activated Jurkat cells and primary T cells. This is consistent with a report showing that infliximab, a monoclonal antibody against TNF-α, increases apoptosis of activated T lymphocytes in the gut mucosa of patients with Crohn's disease [13, 14], although it is possible that infliximab induced apoptosis of activated T cells by antibody-dependent cell-mediated cytotoxicity or complement-dependent cytotoxicity *in vivo*, because our results showed an overexpression of tmTNF-α in activated T cells (Figure 1C and 1G).

We proved for the first time that one of the molecular mechanisms underlying the enhancement of AICD by tmTNF-α-mediated reverse signaling is up-regulating the expression of death ligands FasL, TRAIL and tmTNF-α itself. Nuclear factor kappa B (NF-κB) is one of transcription factors to induce FasL expression [30]. Our previous study demonstrated that NF-κB pathway can be activated by tmTNF-α-mediated reverse signaling [12]. It is not surprising that expression of TNF-α and FasL, as target genes of NF-κB, can be upregulated by treatment with TNF-α pAb. Our results showed that knockdown of expression of FasL or TRAIL blocked the promotion effect of tmTNF-α via reverse signaling on AICD, indicating an amplification effect of tmTNF-α-mediated reverse signaling on AICD.

The second mechanism of reverse signaling of tmTNF-α is up-regulating DR4, both types of TNFR or Fas to increase the sensitivity of activated T cells to AICD. Interestingly, initiation of tmTNF-α reverse signaling significantly increased sTNF-α- and tmTNF-α-mediated apoptosis in activated T cells, however, knockdown of TNFR1 or TNFR2 expression by siRNA abolished the promotion effect of tmTNF-α reverse signaling on sTNF-α- or tmTNF-α-mediated apoptosis, respectively. This indicates that tmTNF-α reverse signaling increases the sensitivity of activated T cells to ACID through upregulating TNFR1 expression to enhance sTNF-α-induced apoptosis and promoting TNFR2 expression to augment tmTNF-α-induced apoptosis. These results point out not only a closely cooperative action between both forms of TNF-α but also a synergistic effect of tmTNF-α-mediated forward signaling and reverse signaling on AICD. Notably, in this experiment target cells were Jurkat cells activated by PHA and TNF-α pAb together for 3 h. Although 3 h-PHA alone activated T cells that expressed tmTNF-α at low level, the reverse signaling of tmTNF-α could be activated by TNF-α pAb during T cell activation. That might promote PHA-induced tmTNF-α expression in T cells, which in turn enhanced the reverse signaling of tmTNF-α to display its effect on AICD.

In summary, our results demonstrate biological activities of tmTNF-α in AICD. tmTNF-α mediates AICD as a ligand via forward signaling and increases the sensitivity of activated T cells to AICD as a receptor via reverse signaling. On the contrary, tmTNF-α reverse signaling also provides a co-stimulatory signal for T cell activation [31, 32]. This transmembrane molecule is similar to other cytokine IL-2 that plays a pivotal role not only in activation of T cells during the early expansion phase after an antigen challenge, but also in controlling AICD through its upregulation of FasL and downregulation of c-FLIP, an anti-apoptotic molecule, during the contraction phase by restimulation [30, 33]. Although the mechanism by which the effect of tmTNF-α can be switched from costimulation in apoptosis-resistant T cells to induction of AICD in apoptosis-sensitive T cells is unclear, targeting tmTNF-α may be beneficial in treatment of autoimmune diseases by promoting AICD of autoreactive lymphocytes.

## MATERIALS AND METHODS

### Reagents

phytohemagglutinin (PHA-P) and Hoechst 33258 were purchased from Sigma-Aldrich; Mouse anti-human CD3 mAb(OKT-3, # 300314) was purchased from Biolegend; Recombinant Human sTNF-α was from Peprotech; Ni^2^+-NTA-Agarose was from Clonetech; TAPI-1(TACE inhibitor, # sc-222337) was purchased from Santa Cruz Biotechnology; Human sTNF-α ELISA kit was from eBioscience; FITC Annexin V Apoptosis Detection kit was purchased from BD Pharmingen; Detoxi-Gel^TM^ Endotoxin Removing Columns (# 20344) was purchased from Thermo Fisher Scientific. The source of antibodies and their catalog numbers were listed in Table 1.

**Table 1:** Names and sources of antibodies used in flow cytometry and Western blot

Name of antibody (usage)	Catalog number	Company
Mouse anti-human TACE (FCM)	ab2051	Abcam
APC anti-human TNFR1 (FCM)	369905	Biolegend
PE anti-human TNFR2 (FCM)	358403	Biolegend
PE anti-human DR5 (FCM)	307405	Biolegend
PE anti-human DR4 (FCM)	307205	Biolegend
Mouse ani-human CD3 mAb (restimulation)	300314	Biolegend
APC anti-human TRAIL(FCM)	sc-56246	Biolegend
Rabbit anti-human β-actin pAb (WB)	AC026	ABclonal
FITC-conjugated goat anti-mouse IgG (FCM)	31561	Thermo Fisher scientific
HRP-conjugated goat anti-rabbit IgG (WB)	62-9520	Thermo Fisher scientific
Rabbit anti-human TNF-α pAb (stimulation or WB)	/	House Made
mouse anti human TNF-α mAb (FCM)	/	House Made
FITC anti-human Fas (FCM)	11-0959	eBioscience
PE anti-human FasL (FCM)	12-9919	eBioscience

FCM: Flow cytometry; WB: Western blot.

### Cell lines and purification of primary human T cells and their stimulation

Jurkat cells, a human acute T lymphocyte leukemia cell line and TNF-α stably transfected NIH3T3 cells [12] were cultured at 37°C in 5% CO_2_ in RPMI-1640 medium supplemented with 10% heat-inactivated pyrogen-free FCS, 1.0 mM sodium pyruvate, 2.0 mM L-glutamine, 100 U/ml penicillin, 100 μg/ml streptomycin. For AICD induction, Jurkat cells were incubated with PHA (5 μg/ml) for 24 h.

Peripheral blood mononuclear cells (PBMC) from healthy volunteers were isolated by Ficoll-Paque (Pharmacia Biotech, Uppsala, Sweden) density centrifugation. Non-adherent cells were collected by adherence of PBMC at 37°C for 1 h. T cells were isolated through nylon columns (Corning, NY, USA), the purity of the CD3^+^ T cells was more than 90% as indicated by flow cytometry. For preactivation, resting T cells were cultured with 5 μg/ml PHA for 3 days. After washing, T cells were cultured for additional 7 days in the presence of 50 U/ml IL-2. The preactivated T cells were restimulated with plate-coated anti-CD3 antibody OKT3 10 μg/ml for 24 h to induce AICD.

### Transfer of AS or siRNAs

The antisense oligonucleotide (AS) used were designed to specifically targeting TNF-α and TACE as previously described [12]. Their targeting sequences were 5′-CTGTGGTACTCGTGACTTTC-3′ and 5′-CAGGAATAGGAGAG ACTGCCT-3′, respectively. Two nonsense oligonucleotides (NS), 5′-CTTTTTGAGC CAGAAGAGGT-3′ and 5′-GATGAGAGAGTC TCCTAGTTG-3′ served as their respective controls. siRNA against human FasL, TRAIL, TNFR1 and TNFR2, and a scrambled control siRNA were designed and synthesized by RiboBio (Guangzhou, China). The sequences used for siRNA targeting were FasL 5′-GGTGGCCTTGTGATCAATG-3′, TRAIL 5′-CTTACGTGTACTTTACCAA-3′, TNFR1 5′-GGAACCUACUUGUACAAUGAC-3′, TNFR2 5′-GGCTCAGAGAATACTATGA-3′. Jurkat or primary human T cells were transfected with 10 μM AS or 100 nM siRNA for 48 h using the Lipofectamine 2000 Reagent (Invitrogen, Carlsbad, CA, USA), according to the manufacturer's instructions.

### RNA isolation and real-time quantitative PCR analysis

Total RNA was extracted from Jurkat cells using TRIzol reagent (Invitrogen, Carlsbad, CA, USA). 2 μg of the total RNA was reversely transcribed to cDNA using Strand cDNA Synthesis SuperMix (TransGen, Beijing, China). Real-time PCR was performed with UltraSYBR Mixture with ROX (CoWinBiotech, Beijing, China) on an Mx3000P Real-time PCR System (Stratagene, la Jolla, CA, USA). The sequences of the primers are as follows: TACE forward: 5′-GTGGATGGTAAAAACGAAAGCG-3′, reverse: 5′-GGCTAGAACCCTAGAGTCAGG-3′; TRAIL for-ward: 5′-TGCGTGCTGAT CGTGATCTTC-3′, reverse: 5′-GCTCGTTGGTAAAGTACACGTA-3′); FasL forward: 5′-TGCCTT GGTAGGATTGGGC-3′, reverse: 5′-GCTGGTAGACTCTCGGAGTTC-3′; and β-actin forward: 5′-AGTTGCGTTACACCCTTTC-3′, reverse: 5′-CACCTT CACCGTTCCAGT- 3′. The PCR reaction was performed in triplicate and the following thermal cycling condition was used: 10 min at 95°C, followed by 15 s at 95°C, 1 min at 60°C for 40 cycles. Results were analyzed with Stratagene Mx3000P software using the 2-^△△Ct^ method and normalized with β-actin.

### sTNFR 1 expression and purification

Human cDNA coding for the extracellular region of sTNFR1 (275-772) was constructed and cloned into the pET-28a (+) vector at the Bam HI and Hind III cloning site [11]. sTNFR 1 was expressed in *Escherichia coli* upon stimulation with 1 mM IPTG, and purified using a Ni2^+^-NTA resin. The purity was 95%. Endotoxin was removed with a Detoxi-Gel endotoxin-removing column according to the manufacturer's instructions. Residual endotoxin concentration was <0.2 U/mg.

### Flow cytometry

Cells were collected after stimulation and washed by pre-cold PBS for 3 times. The PE, APC or FITC-conjugated antibodies or unconjugated primary antibodies were then added and incubated at 4°C for 1 h. The incubation with primary antibodies was followed by staining at 4°C for 45 min with FITC-conjugated secondary antibody. The expression of tmTNF-α, Fas, FasL, TRAIL, DR4, DR5, TNFR1 and TNFR2 was analyzed on a FACS Calibur 440E flow cytometer (Becton Dickinson, San Jose, CA, USA).

### Apoptosis detection

The apoptosis was evaluated by an Annexin V-FITC Apoptosis Detection Kit (BD biosciences), according to the manufacturer's instructions. Briefly, cells after stimulation were collected, washed twice with precold PBS and resuspended in 100 μl binding buffer. 5 μl of Annexin V-FITC and 10 μl of PI (50 μg/ml) were added into the suspension. Cells were then stained for 15 min at room temperature (RT) in the dark. Apoptosis was analyzed by flow cytometry. Apoptosis (%) = percentage of Annexin V positive cells + percentage of both Annexin V and PI positive cells.

For Hoechst 33258/PI double staining assay, primary human T cells after activation or reactivation were stained for 7 min at 37°C with Hoechst 33342 (5 μg/ml), then followed by PI (1 μg/ml) for 7 min at RT. Then the stained cells were observed under a fluorescence microscope (Nikon DXM1200 fluorescence microscope, Japan).

### ELISA for sTNF-α

The concentration of sTNF-α in supernatants was determined by a Human TNF-α ELISA kit, according to the manufacturer's instructions. Briefly, the supernatant was collected after activation of T cells. A human monoclonal antibody specific to TNF-α was used to coat ELISA plates. After incubation with samples and the standard of TNF-α at RT for 2 h, abiotin-conjugated monoclonal anti-human TNF-α antibody was added and cultured for 1 h at RT, followed by the incubation with streptavidin-HRP for 30 min after washing. The color was developed for 15 min by addition of TMB substrate solution and the absorbance was measured at 450 nm on a microplate reader (Tecan, Groedig, Austria).

### TNF-α Bioassay

sTNF-α Bioassay: 2 x10^5^ Jurkat cells were incubated with 5 μg/ml of PHA-P or/and 50 U/ml of sTNF-α for 24 h. 2 x10^5^ PHA-preactivated primary T cells were reactivated for 24 h with αCD3 (10 μg/ml) in the absence or presence of 50 U/ml of sTNF-α. sTNF-α-mediated apoptosis was measured by Annexin V/PI.

tmTNF-α Bioassay: Jurkat or preactivated primary T cells was activated or reactivated with 5 μg/ml of PHA-P or α-CD3 mAb (10 μg/ml) for 24 h, respectively. These tmTNF-α overexpressing cells or TNF-α stably transfected NIH3T3 cells were used as effector cells and fixed in 1% paraformaldehyde. To remove receptor-bound sTNF-α, cells were incubated with acid glycine buffer (Gly-NaCl, pH 3.0) for 15 min after fixation, then washed twice with PBS. 1×10^6^ effector cells were adhered to polylysine-coated microplate and air dried. 1 x10^5^ 3 h-PHA activated Jurkat cells or preactivated primary T cells as target cells were added to each well that contained effector cells adherent to polylysine and incubated for 48 h. tmTNF-α-induced apoptosis was determined by Annexin V/PI.

### Western blot

Total protein was extracted by lysis of cells in pre-cold buffer A (10 mM HEPES, pH 7.8, 10 mM KCl, 0.1 mM EDTA, 1mM DTT) and a protease inhibitor cocktail (Sigma-Aldrich, St. Lous, MO, USA) on ice for 20 min. After centrifugation at 12,000 x g for 20 min at 4°C, the total protein was collected. 50 μg of protein was electrophoresed on a SDS-polyacrylamide gels and transferred to PVDF membranes (Millipore, Billerica, MA, USA) using a semi-dry transfer system (BioRad Laboratories, Hercules, CA, USA). The membranes were blocked for 2 h at RT with 5% non-fat dry milk in PBS containing 0.05% Tween-20 and then probed overnight at 4°C with primary antibodies including anti-TNF-α and anti-β-actin, followed by horseradish peroxidase-conjugated anti-rabbit IgG secondary antibody at RT for 1 h. The bands were visualized using SuperSignal West Pico Chemiluminescence Substrate (Thermo, Waltham, MA, USA).

### Statistical analysis

One- or two-way analysis of variance (ANOVA) was used for statistical analysis. Data are represented as mean ± S.D. *p* < 0.05 is considered to be statistically significant.
